# State-Related Alterations of Spontaneous Neural Activity in Current and Remitted Depression Revealed by Resting-State fMRI

**DOI:** 10.3389/fpsyg.2019.00245

**Published:** 2019-02-11

**Authors:** Chang Cheng, Daifeng Dong, Yali Jiang, Qingsen Ming, Xue Zhong, Xiaoqiang Sun, Ge Xiong, Yidian Gao, Shuqiao Yao

**Affiliations:** ^1^Medical Psychological Center, The Second Xiangya Hospital, Central South University, Changsha, China; ^2^Preschool Education Department, Changsha Normal University, Changsha, China; ^3^Department of Psychiatry, The First Affiliated Hospital of Soochow University, Soochow University, Suzhou, China

**Keywords:** major depressive disorder, remission, trait-related, state-related, resting-state fMRI, amplitude of low-frequency fluctuation

## Abstract

**Purpose:** Although efforts have been made to identify neurobiological characteristic of major depressive disorder (MDD) in recent years, trait- and state-related biological characteristics of MDD still remains unclear. Using functional magnetic resonance imaging (fMRI), the aim of this study was to explore whether altered spontaneous neural activities in MDD are trait- or state- related.

**Materials and Methods:** Resting-state fMRI data were analyzed for 72 current MDD (cMDD) patients (first-episode, medication-naïve), 49 remitted MDD (rMDD) patients, and 78 age- and sex- matched healthy control (HC) subjects. The values of amplitude of low-frequency fluctuation (ALFF) were compared between groups.

**Results:** Compared with the cMDD group, the rMDD group had increased ALFF values in the left middle occipital gyrus, left middle temporal gyrus and right cerebellum anterior lobe. Besides, compared with the HC group, the cMDD group had decreased ALFF values in the left middle occipital gyrus. Further analysis explored that the mean ALFF values in the left middle occipital gyrus, left middle temporal gyrus and right cerebellum anterior lobe were correlated positively with BDI scores in rMDD patients.

**Conclusion:** Abnormal activity in the left middle occipital gyrus, left middle temporal gyrus and right cerebellum anterior lobe may be state-specific in current (first-episode, medication-naïve) and remitted (medication-naïve) depression patients. Furthermore, the state-related compensatory effect was found in these brain areas.

## Introduction

Major depressive disorder (MDD) is a high-recurrence psychiatric condition. which more than half of patients seen for first-episode MDD in China experience a recurrence of MDD symptoms within 5 years after the initial depression onset (Ji et al., 2001). Moreover, clinical factors, including the number of previous episodes and subclinical residual symptoms, appear to be the most important predictors of recurrence (Hardeveld et al., 2010). Therefore, distinguishing the correlation between altered brain activity and different clinical states may lead to a better understanding of the neurobiological mechanisms underlying MDD pathogenesis and recurrence, which, in turn, may improve clinical diagnosis and prognostic evaluation of MDD.

Some neuroimaging studies, primarily using task-related functional magnetic resonance imaging (fMRI) and structural magnetic resonance imaging (sMRI) methods, have examined brain activation and structural changes in different clinical states, including current MDD (cMDD) and remitted MDD (rMDD). These researches have explored several abnormal alterations of brain region in patients, which could be trait-related or state-related markers of depression. However, a number of brain regions, including regions within the orbitofrontal cortex, insular cortex, posterior cingulate cortex (PCC), amygdala, hippocampus, and prefrontal cortex, have been dually implicated as potential trait-related and state-related biomarkers of depression (Caetano et al., 2004; Wang et al., 2008; Lorenzetti et al., 2010; van Eijndhoven et al., 2013; Liu et al., 2014; Ming Q. et al., 2017). Inconsistency across studies might be partially related to different task paradigms or analytical methods, including stress task or source recollection paradigm, cortical thickness or brain region volume analysis.

Resting-state fMRI studies, wherein subjects are not performing an explicit task during the scan, can complement task fMRI studies (Su et al., 2010; Biswal, 2012). Resting-state data can be analyzed by multifarious approaches, such as seed-based approaches, independent component analysis, graph methods, clustering algorithms, neural networks, and pattern classifier (Lee et al., 2013; Dong et al., 2018). In recent years, a resting-state fMRI analytical method named amplitude of low-frequency fluctuation (ALFF) was developed to assess the spontaneous low frequency (0.01–0.08 Hz) fluctuations (LFF) in the BOLD fMRI signal at rest, which could reflect the intensity of brain regional spontaneous neural activity (Zang et al., 2007). Besides, some articles indicated that the ALFF was associated with the neuronal glucose metabolism and local field potential activity (Logothetis et al., 2001; Tomasi et al., 2013). Therefore ALFF analysis has been widely used to explore potentially related cerebral biomarkers in mental disorders, which was shown to be a reliable and sensitive approach (Zhang et al., 2014; Fan et al., 2017).

To our knowledge, only one resting-state fMRI study investigating state- and trait-related functional alterations in cMDD and rMDD have been published. Jing reported that abnormal activity of the putamen may be a potential trait-related marker of MDD (Jing et al., 2013). However, Jing’s study included only female patients and did not exclude the effects of comorbidities, treatment condition, or the number of prior episodes. Some depression researches indicated that the comorbidities, treatment condition, and the number of prior episodes would make a difference in the activities of brain areas, such as cingulate cortex, prefrontal lobe, striatum, temporal lobe and insula (Schaefer et al., 2006; Takami et al., 2007; Waugh et al., 2012). Thus the effects of comorbidities, treatment condition, the number of prior episodes should be considered in the depression study.

Notably, Mayberg’s classical neurobiological model implied that the MDD patient has fronto-limbic dysfunctions, including the prefrontal cortex, cingulate cortex, amygdala and striatum, which would account for the dysregulation of the affective and cognitive behavior in patients (Mayberg, 1997, 2003). Moreover, some meta-analysis articles of resting-state fMRI suggested that the dysfunctions of the fronto-limbic circuit, default mode network (DMN) and cerebellum, which play an important role in the cognitive processing and affective regulation, would be the biomarkers of drug-naive MDD patients (Xue et al., 2016; Ming Z.M. et al., 2017).

The purpose of this study was to investigate MDD state-related and trait-related neuroimaging alterations of spontaneous neural activity by resting-state fMRI. To exclude the potential influence of comorbidities, treatment condition, and the number of prior episodes, we enrolled a large sample, including 72 first-episode, medication-naive MDD patients (cMDD), 49 remitted MDD patients (rMDD), and 78 age- and sex- matched healthy controls (HCs). The participants were subjected to resting-state fMRI with ALFF analysis. In the context of classical neurobiological model and the studies mentioned above, we hypothesized that the state-related or trait-related characteristic of MDD would be explored in the brain areas of fronto-limbic circuit, default mode network and cerebellum.

## Materials and Methods

### Participants

The cMDD and rMDD participants were recruited from the psychology clinic at Second Xiangya Hospital affiliated with Central South University in Changsha, Hunan, China. With advertisements and posters, age-, sex-matched HCs were recruited from two colleges and a local community in Changsha. The sample of all the depression patents and normal people was registered from 2014 to 2017.

The clinical states of the patients, including cMDD and rMDD groups, were evaluated independently by two psychiatrists using the Structured Clinical Interview for DSM-IV-TR Axis I Disorders-Patient Edition (First et al., 2002) and 17-item Hamilton Depression Rating Scale (Hamilton, 1960). At the same time, demographic data and clinical variables information were collected by interview. Patients, who met the DSM-IV-TR criteria for MDD and were in their first MDD, were included in the cMDD group. Inclusion criteria for the rMDD patients were as follows: having at least one episode of MDD in the past 10 years; not meeting the DSM-IV-TR criteria for MDD more than 30 days before the scan; a 17-item HAM-D score ≤7 on the scan day. In this study the remitted MDD was not described as clinical outcome of recovery but a remitted state, which was not shown currently presenting symptoms of MDD. Moreover, this criterion has been used in previous studies of remitted depression (Goulden et al., 2012). The HC group was required to have no history of any prior DSM-IV-TR Axis I disorder. The exclusion criteria for all three groups were: a history of alcohol/substance abuse; use of antidepressants or undergoing psychotherapy or psychotropic medications; having other major psychiatric disorder; a neurological disorder diagnosis; structural brain abnormalities; and any MRI contraindication.

All participants were informed of the study’s purpose and signed informed consent forms. This study was approved by the Ethics Committee of the Second Xiangya Hospital of Central South University.

### Psychological Measures

All participants filled out a Beck Depression Inventory-II (Beck et al., 1996) composed of 21 self-report items, which proved to be a validated depressive symptoms scale. This multiple-choice inventory could evaluate the MDD symptoms including irritability, feelings of guilt, suicidal ideation, fatigue, and weight loss (Ayloo et al., 2015). In our current sample, Cronbach’s alphas for cMDD, rMDD, and HC groups were 0.850, 0.858, and 0.823, respectively.

### Image Acquisition

Magnetic resonance imaging (MRI) scans were performed on a 3.0-T Siemens Magnetom Skyra scanner (Siemens Healthineers, Erlangen, Germany). During scanning, all participants were instructed to remain motionless with their eyes closed, and to think of nothing in particular but to not fall asleep. To reduce patient head movements and noise, the subjects were fit with foam pads about the head and earplugs. The acquisition parameters were as follows: 32 axial slices, 4-mm slice thickness with a 1-mm gap, 2000-ms repetition time, 30-ms echo time, 80° flip angle, 256 × 256-mm field of view, 64 × 64 data matrix. Three-dimensional T1-weighted magnetization-prepared rapid gradient echo sagittal images were also acquired with the following parameters: 176 axial slices with no gap, 1900-ms repetition time, 2.01-ms echo time, 1-mm^3^ voxel size, 9° flip angle, 256 × 256-mm field of view, 256 × 256 data matrix.

### Data Processing

Resting-state fMRI data preprocessing was conducted in Data Processing Assistant for Resting-State fMRI software (DPARSF V2.3, Yan and Zang, 2010^1^). The first 10 volumes of the functional time series were removed to ensure stable magnetization and adaptation of participants to scanning noise. Subsequently, slice timing and head motion correction were performed. Data were discarded from 30 subjects (10 cMDD, 9 rMDD, and 4 HC) due to translation >2 mm in any direction or rotation >2° around any axis in any of six head motion parameters. Besides, regression of the Friston 24 motion parameters was conducted to control the potential influence of head motion (Satterthwaite et al., 2013). After the correction mentioned above, the EPI template of standard Montreal Neurological Institute (MNI) was used for spatial normalization with a resampling voxel size of 3 × 3 × 3 mm^3^. The preprocessing image was spatially smoothed with an 8 × 8 × 8 mm full width at half-maximum Gaussian kernel.

To discard biases from low-frequency drift and high-frequency noise, detrending and band-pass (0.01–0.08 Hz) filtering were conducted. After that, the time series of each voxel was converted into the frequency domain, of which power spectrum was calculated. The square root was calculated at each frequency of the power spectrum. Finally, the average square root was then obtained at each voxel across the frequency range of 0.01–0.08 Hz, which was obtained to take as ALFF to reflect absolute intensity of brain spontaneous neural activity (Zang et al., 2007).

After the data processing, the final analysis included 72 medication-naive, first-episode cMDD patients (39 females), 49 rMDD patients (26 females), and 78 HC subjects (43 females).

### ALFF Data Statistical Analysis

Amplitude of low-frequency fluctuations maps were processed using the Resting-State fMRI Data Analysis Toolkit (REST V1.8, Song et al., 2011; see text footnote^1^). These maps were then exported to SPM12 (Wellcome Trust Center for Neuroimaging, London, United Kingdom^2^) for statistical analyses. An analysis of covariance (ANCOVA) was used to detect between group differences on ALFF maps, including levels of education as a covariate of no interest. An initial voxelwise threshold of *p* < 0.001 uncorrected was used, to which a clusterwise correction (FDR) for multiple comparison was applied.

Independently of the result, the map from the uncorrected main effect of group from the ANCOVA was used to mask the exploratory between group comparisons among the three different groups (Henson et al., 2002; Fan et al., 2017). For these exploratory analyses, an initial voxelwise threshold of *p* < 0.001 uncorrected was used, to which a clusterwise correction for multiple comparison (FDR) was applied. An additional Bonferroni correction for multiple comparisons was applied on the cluster *p*-value to account for the number of tests performed (6 comparisons: cMDD > rMDD, cMDD < rMDD, cMDD > HC, cMDD < HC, rMDD > HC, rMDD < HC). A cluster with a *p*-value of 0.008 or below would therefore be significant (*p* = 0.05/6 = 0.008).

To further examining the connection between the abnormal regional brain activity and depression symptom severity, correlation analyses were conducted between psychometric (HAM-D and BDI) scores and mean ALFF values obtained in brain regions with abnormal activity in the cMDD and rMDD groups, separately.

## Results

### Cohort Characteristics

The demographic and clinical data are summarized in Table 1. The groups’ characteristics did not differ in terms of sex or age, though the HC group’s education level was, on average, higher than that the other two groups [HC > cMDD = rMDD, *F*(2,196) = 12.023, *p* < 0.001]. Mean HAMD scores were greater in the cMDD group than in the rMDD group (*t* = 14.410, df = 119, *p* < 0.001). A one-way ANOVA revealed a main effect of group on BDI scores [*F*(2,196) = 310.668, *p* < 0.001] and *post hoc* analysis showed that each group’s BDI scores differed from those of the other two (cMDD > rMDD > HC, all *post hoc p*-values < 0.005).

**Table 1:** Demographic and clinical characteristics of cMDD, rMDD, and HC groups^a^.

Characteristic	cMDD (*N* = 72)	rMDD (*N* = 49)	HC (*N* = 78)	*F*/*t*	*P*	$\eta_{p}^{2}$ / Cohen’s d
Age, years	22.38 (5.67)	22.57 (6.41)	22.19 (3.52)	0.083	0.921	<0.001
Sex, N females (%)	39 (54.2)	26 (53.1)	43 (55.1)	0.049	0.952	<0.001
Education, years	13.28 (2.51)	13.87 (2.48)	15.10 (1.98)	12.023	<0.001	0.123
Illness duration, years	0.85 (0.83)	1.22 (0.92)	–	1.571	0.102	0.42
Remission duration, years	–	0.51 (0.30)	–	–	–	–
HAM-D score	22.25 (6.00)	6.45 (5.81)	–	14.410	<0.001	2.68
BDI score	29.46 (9.32)	6.91 (5.87)	3.23 (3.99)	310.668	<0.001	3.17

*Means are shown with standard deviations in parentheses, unless otherwise specified. ^*a*^cMDD, current major depressive disorder; rMDD, remitted major depressive disorder; HC, healthy control; BDI, Beck Depression Inventory; HAM-D, 17-item Hamilton Depression Rating Scale.*

### ALFF Differences

**FIGURE 1:**
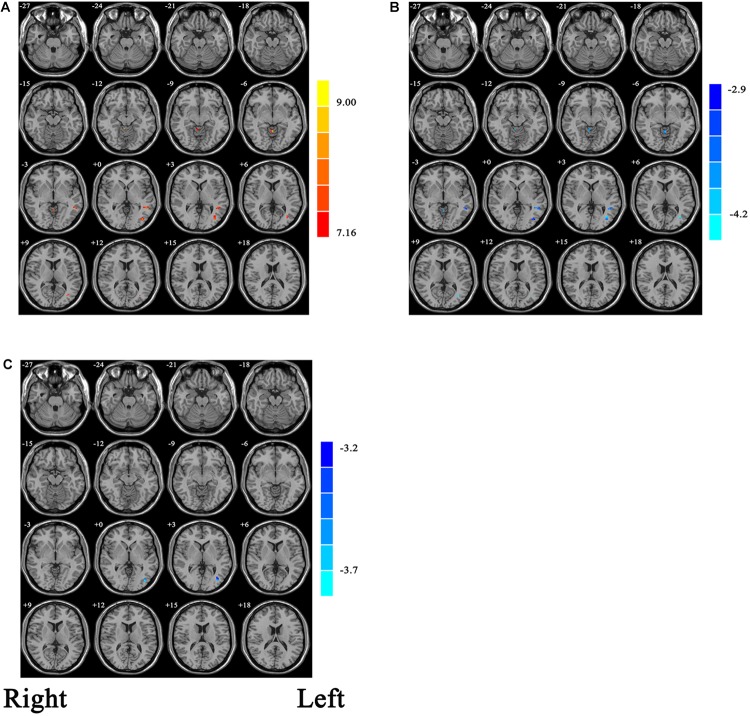
**(A)** Statistic maps showing ANOVA results of ALFF differences among current major depression disorder (cMDD), remitted major depression disorder (rMDD), and healthy control (HC) groups (*p* < 0.001, uncorrected). **(B)** Brain regions showing ALFF differences between cMDD and rMDD [*p* < 0.008, false discovery rate (FDR) corrected]. **(C)** Brain regions showing ALFF differences between cMDD and HC (*p* < 0.008, FDR corrected). ANOVA and *post hoc t*-tests were conducted using years of education as covariates of no interest. Two-sample *t*-test results are expressed within a mask showing significant group differences from the ANOVA. Red and blue denote ALFF increases and decreases, respectively; color bars indicate *t*-values.

With the one-way ANCOVA analysis, main effects of group on ALFF values were identified in the brain areas including the occipital and temporal cortices, as well as in the cerebellum (*p* = 0.001, uncorrected; see Figure 1A).

Within the mask of these significant group differences, *post hoc t*-tests showed that, compared with the cMDD group, rMDD group had increased ALFF values in the left middle occipital gyrus, left middle temporal gyrus and right cerebellum anterior lobe (Table 2 and Figure 1B). Besides, compared with the HC group, the cMDD group had decreased ALFF values in the left middle occipital gyrus (Table 2 and Figure 1C). Furthermore, all *p*-values in the *post hoc t*-tests were corrected with false discovery rate (FDR) method (all *p*-values < 0.008; see Table 2).

**Table 2:** Brain regions with significantly different ALFF values among the cMDD, rMDD, and HC groups.

Brain regions	Voxels	Peak coordinates (MNI)			Peak *T*-values	*P* uncorrected	*P* corrected^a^
		*x*	*y*	*z*			
**cMDD < rMDD**							
Left middle occipital gyrus (BA 19)	17	−36	−72	6	−4.5577	0.001	0.004
Left middle temporal gyrus	15	−45	−45	3	−3.8307	0.001	0.004
Right cerebellum anterior lobe	11	6	−45	−12	−4.2424	0.001	0.004
**cMDD < HC**							
Left middle occipital gyrus (BA 19)	12	−39	−78	0	−3.8093	0.001	0.002

*ALFF, amplitude of low frequency fluctuations; BA, Brodmann area; x, y, z, coordinates of peak locations in the Montreal Neurological Institute (MNI) space. ^a^False discovery rate (FDR) corrected (p < 0.008).*

### Correlations Between ALFF Values and Clinical Variables

**FIGURE 2:**
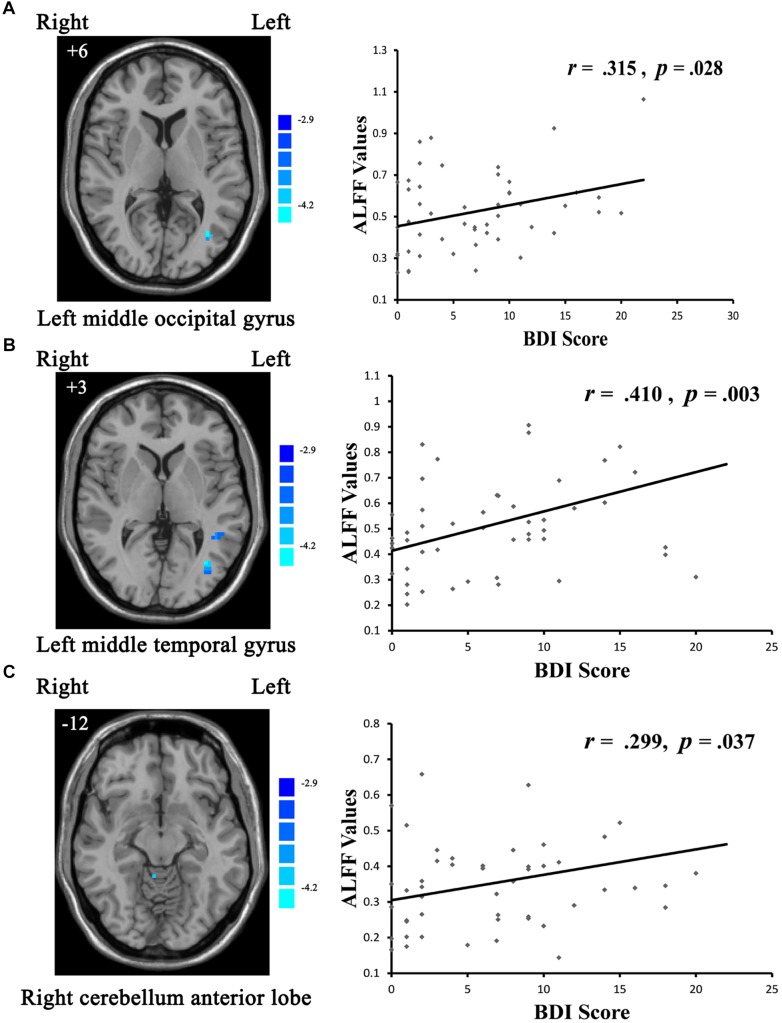
Scatter plots showing significant positive correlations between BDI scores and regional ALFF values in the **(A)** left middle occipital gyrus (uncorrected *p* = 0.028), **(B)** left middle temporal gyrus (uncorrected *p* = 0.003; significant with Bonferroni correction), **(C)** right cerebellum anterior lobe (uncorrected *p* = 0.037).

With respect to potential relationships between regional brain activity and clinical variables, mean ALFF values in the cMDD and rMDD groups, were separately obtained in the brain regions showing differences with the one-way ANCOVA analysis. The correlations between the mean ALFF values and BDI, HAMD scores were examined. Eventually, only the BDI scores in the rMDD group were correlated positively with ALFF values in the left middle occipital gyrus (*r* = 0.315, uncorrected *p* = 0.028; see Figure 2A), left middle temporal gyrus (*r* = 0.410, uncorrected *p* = 0.003; significant with Bonferroni correction [0.05/6]; see Figure 2B), right cerebellum anterior lobe (*r* = 0.299, uncorrected *p* = 0.037; see Figure 2C).

## Discussion

The major finding of the current study was that the rMDD group showed increased hyperactivities in the left middle occipital gyrus, left middle temporal gyrus and right cerebellum anterior lobe when compared with the cMDD group by resting-state fMRI. Compared with the HC group, the cMDD group demonstrated decreased hypoactivity in the left middle occipital gyrus. The spontaneous neural activities in the left middle occipital gyrus, left middle temporal gyrus and right cerebellum anterior lobe were positively relevant with clinical symptom. These results suggested that brain activities in the left middle temporal gyrus, left middle occipital gyrus, and right cerebellum anterior lobe may serve as state-related biological characteristics of MDD.

The middle temporal gyrus, which is located between the superior temporal gyrus and inferior temporal gyrus, plays a vital role in the cognitive processing, such as language, memory and object vision processing (Onitsuka, 2004). Colombo and Gross (1994) reported that deficit in the middle temporal gyrus in monkeys would cause a poor performance in the cognitive task which requires visual object discrimination and recognition. A structural MRI study reported gray matter reduction in the middle temporal gyrus in treatment-resistant depression patients when compared with healthy subjects (Ma et al., 2012). Besides, the middle temporal gyrus is involved in the DMN (Zhou and Yu, 2010; Xue et al., 2016). Abnormal DMN functional alterations have been found closely bound up with rumination and autobiographical memory impairment in depression patients (Sumner et al., 2010; Paul et al., 2015).

The present study also found more hyperactivity in the middle temporal gyrus in the rMDD group compared with the cMDD group. This result and the relation between clinical symptom and brain activity in rMDD group may indicate the state-related functional compensation in the middle temporal gyrus. The functional compensation implies that the individuals suffered from damage of the central nervous system (CNS) would trigger the residual structures to achieve recovery, including behavioral, physical, or cognitive strategies (Tanaka, 2001). Furthermore, the temporal regions were deeply involved in social cognitive and affective processing (Wang et al., 2012). Therefore, we tentatively put forward that the state-related hyperactivity in the middle temporal gyrus may be involved in a compensatory mechanism, which is consistent with Goetz’s study that the compensatory effect has been found in the same brain region among the depression patients during a cognitive reappraisal task (Goetz et al., 2018).

Consistent with previous studies (Guo et al., 2013; Liang et al., 2013), the hyperactivity in the left middle occipital gyrus was found in MDD patients when compared with HC. The brain activity level in the left middle occipital gyrus was positively relevant with clinical symptom. The depressed patients with occipital brain activity abnormalities have shown a disproportionate attentional preference toward negative visual information (Guo et al., 2013). Depression-associated abnormalities of the middle occipital gyrus were related with abnormal neuropsychological activity, leading to a motor block and lowered attention (Yu et al., 2017). As described previously, this increased brain activity may be due to the state-related compensatory effect in the left middle occipital gyrus, which plays an active role in cognitive processing, including visual information processing and verbal episodic memory (Li et al., 2016).

The cerebellum, a structure used to be most appreciated for its important role in motor coordination and behavior (Stein, 1986), was reported taking part in emotional and cognitive processing in recent depression studies (Schmahmann, 2000; Lekeu et al., 2002; Lin et al., 2012). Our study found increased activities in the anterior lobe of cerebellum in the rMDD group than in the cMDD group. Besides, the activity level in this area was correlated with depressive symptom in the rMDD group, which may imply that the state-related hyperactivities in the anterior lobe of cerebellum found in the rMDD patients are involved in a compensatory mechanism. The relation between cerebellum brain activity and depressive symptom has been reported by fMRI and sMRI findings (Zeng et al., 2012; Xu et al., 2017). This association may be illustrated by the cerebellar connections with limbic regions, brainstem, temporal lobe, prefrontal lobe, and cingulate gyrus, areas shown to have profound influence on cognitive processing and emotional regulation (Haines et al., 1984; Middleton and Strick, 2001; Turner et al., 2007). A growing number of studies have paid attention to the role of cerebellum in depression (Lin et al., 2012; Phillips et al., 2015; Ming Z.M. et al., 2017), and our research implied that the anterior cerebellum may be related with the depression remitted process.

### Limitations

Our study has some limitations that need to be addressed in further studies. Firstly, we used a cross-sectional study design. Longitudinal studies are required to clarify how neural brain activities evolve from depression onset to remission, and from remission to recurrence. Second, to exclude potential confounders, we excluded patients with comorbidities. However, more than three-quarters of MDD patients have comorbidities (Kessler et al., 2003). Thus, it is not known whether the state-related features found in this study would be detectable in MDD patients with comorbidities. Third, in our study, the ANCOVA anlysis result of ALFF values was uncorrected and voxels cluster size was small for a clusterwise correction. Therefore, our findings, as an exploring research, may only imply a trend of the abnormal activity brain area in cMDD, rMDD and HC groups. Longitudinal studies across different clinical states would be required in the future exploration.

## Conclusion

To our knowledge, this is the first study to explore state-related alterations of spontaneous neural activity in medication-naïve current and remitted depression. Consistent with the research hypothesis, state-related abnormal spontaneous neural activities were observed in the DMN and cerebellum, including the middle temporal gyrus, cerebellum anterior lobe. Furthermore, the state-related compensatory effect was found in the middle occipital gyrus, middle temporal gyrus and cerebellum anterior lobe. Although these findings remain to be confirmed, our study provides a fresh perspective for elucidating the neurobiology of MDD development, maintenance and recovery. The state-related characteristic of MDD suggested by our study may be useful for improving clinical diagnosis and prognostic evaluation of MDD, planning of targeted interventions, and monitoring of therapeutic efficacy.

## Author Contributions

SY supervised the study. CC performed the analysis and wrote paper. DD, YJ, and QM contributed to the analysis. XZ, XS, GX, and YG collected the data. All co-authors revised and approved the version to be published.

## Conflict of Interest Statement

The authors declare that the research was conducted in the absence of any commercial or financial relationships that could be construed as a potential conflict of interest.

## Footnotes

1 http://www.restfmri.net

2 http://www.fil.ion.ucl.ac.uk/spm
